# Studying bacterial transcriptomes using RNA-seq

**DOI:** 10.1016/j.mib.2010.09.009

**Published:** 2010-10

**Authors:** Nicholas J Croucher, Nicholas R Thomson

**Affiliations:** The Wellcome Trust Sanger Institute, Wellcome Trust Genome Campus, Hinxton, Cambridge, Cambridgeshire, CB10 1SA, UK

## Abstract

Genome-wide studies of bacterial gene expression are shifting from microarray technology to second generation sequencing platforms. RNA-seq has a number of advantages over hybridization-based techniques, such as annotation-independent detection of transcription, improved sensitivity and increased dynamic range. Early studies have uncovered a wealth of novel coding sequences and non-coding RNA, and are revealing a transcriptional landscape that increasingly mirrors that of eukaryotes. Already basic RNA-seq protocols have been improved and adapted to looking at particular aspects of RNA biology, often with an emphasis on non-coding RNAs, and further refinements to current techniques will improve our understanding of gene expression, and genome content, in the future.

## Introduction

The advent of second generation sequencing technologies has created many opportunities to improve functional genomics experiments, including quantitative gene expression studies. Most previous transcriptional analysis methods have relied on hybridization of targeted oligonucleotides to particular loci for their sequence specificity: either primers binding to target cDNA in quantitative reverse transcription polymerase chain reaction (qRT-PCR), labeled probes binding to RNA in Northern blotting or hybridization of cDNA to probes on microarray chips. RNA-seq is different in principle in that data are matched to genes by sequence alignment instead.

This has intrinsic advantages: first, because no probe sequences are specified, all transcription is studied in an unbiased manner, and experimental design does not need to be altered in accordance with differences in genome sequence. This promises to be a particular advantage in the study of bacteria with large amounts of genetic variation between strains [1]. It also allows the discovery of novel genetic features, as well as permitting the delineation of operons and untranslated regions, allowing the improvement and extension of sequence annotation.

Second, mapping of sequence data is more precise than hybridization between oligonucleotides. This allows transcription to be studied at a much higher resolution by sequencing, thereby also permitting the study of more repetitive regions of the genome. Additionally, it means quantification of gene expression by RNA-seq does not suffer from the issues of interference between genes due to non-specific hybridization of cDNA to probes [2,3].

Third, whereas hybridization-based methods measure gene expression levels through detection of fluorescence or radioactivity, RNA-seq uses the amount of data matching a given coding sequence (CDS), typically quantified as reads per kilobase CDS length per million reads analyzed (RPKM) [4]. This measure cannot be saturated in the way the detection of light or radioactivity can, hence RNA-seq has a much greater dynamic range for measuring variability in expression levels. Consequently, it can also be much more discriminatory both at high levels of gene expression and more sensitive at very low levels of expression, given sufficient sequencing depth.

## Preparation of cDNA

RNA is typically extracted using organic solvents or commercially available kits; however, care should be taken to ensure the method does not bias the sampling of the transcriptome [5] and is capable of harvesting sufficient starting material needed to construct a sequencing library, as more RNA is typically needed than for microarray experiments. Furthermore, the exclusion of highly expressed transcripts, which risk saturating the dataset, is also more difficult than with microarray experiments, where probes can be omitted from the chip design as required. As ribosomal RNA comprises the vast majority of the extracted RNA population, depletion of these molecules through hybridization to magnetic bead-linked complementary oligonucleotides [5–10,11^•^], or the use of terminator exonucleases that specifically degrade transcripts with a 5′ monophosphate group [12^••^], has been used in efforts to increase the coverage of mRNA and ncRNA. However, the rapid increase in the productivity of the second generation sequencing technologies renders the expensive depletion processes largely unnecessary, especially given the opportunity for sample degradation and bias it presents [10]. Nevertheless, saturation of sequence data by abundant transcripts will remain an issue in some cases; for instance, when analyzing bacterial gene expression within host tissues, where eukaryotic RNA will be far more abundant than that of the prokaryote.

**Figure 1:**
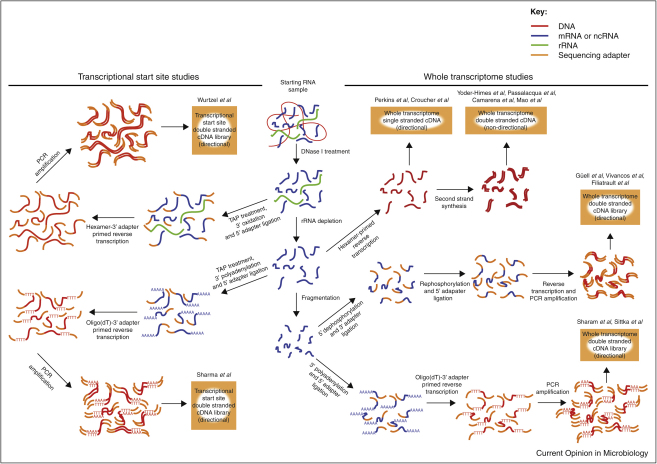
Methods for preparation of cDNA. All methods require the extraction of nucleic acids from a sample of cells, followed by the enzymatic removal of DNA. Ribosomal RNA may then be depleted to increase the sequence coverage of other transcripts. To identify putative transcriptional start sites, samples are first treated with tobacco acid pyrophosphatase (TAP), which converts the triphosphate group at the 5′ end of intact transcripts to a monophosphate. This is required for the ligation reaction to attach an adapter to the 5′ end; polyadenylation or oxidation of the 3′ end of the RNA is used to ensure the specificity in the orientation of this reaction. This allows the 3′ part of the cDNA, corresponding to the extreme 5′ end of the original transcript, to be targeted for sequencing. In order to obtain sequence data covering the entire transcriptome, small cDNA molecules must be randomly generated from throughout the RNA sample. This has frequently been achieved through random hexamer-primed reverse transcription; using only the first strand for sequencing library construction allows information on the direction of transcription to be maintained. Alternatively, the RNA may be fragmented, and information on the template strand for transcription retained through orientation-specific, stepwise attachment of adapters. One method involves dephosphorylating the 5′ end so the first adapter can only be ligated to the 3′ end of the transcript; the complementary approach is to polyadenylate the 3′ end such that the first adapter is only found attached the 5′ end of the RNA. One technique not shown is the use of fragmented RNA as a template for random hexamer-primed reverse transcription, as performed by Oliver *et al.* A wider range of methods has been applied in obtaining similar information from eukaryotic transcriptomes (see text).

In the original RNA-seq protocols, following extensive DNase treatment, RNA was typically converted into cDNA through random hexamer-primed reverse transcription followed by second DNA strand synthesis [5–9,13^•^]. However, using double stranded cDNA for making sequencing libraries results in equal levels of signal on both the sense and antisense strands, thereby losing information regarding the direction of transcription. A simple method for maintaining the directional signal in RNA-seq data is to construct Illumina libraries from only first strand cDNA [10]. Alternative techniques used to maintain directional fidelity involve sequentially ligating adapters onto RNA molecules in an orientation-specific manner [14,15], with one approach implemented in studies of *Mycoplasma pneumoniae* and *Pseudomonas syringae* transcriptomes [16^••^,17^•^] and another used for RNA-seq in *Helicobacter pylori* and *Salmonella enterica* Typhimurium [12^••^,18^•^] (Figure 1). Other methods for maintaining directional information pioneered in studies of eukaryotes include the use of template switching PCR [19], bisulfite-induced conversion of cytosine to uracil in transcripts before reverse transcription [20], addition of sequence tags into the primers used for reverse transcription [21] and incorporation of deoxyuridine into the second strand of cDNA, which can subsequently be degraded using uracil-*N*-glycosylase [22]. The importance of this information in characterizing ncRNA and observing antisense transcription is becoming increasingly evident.

## Alternative applications of RNA-seq

As well as surveying the entire transcriptomes of bacterial strains, RNA-seq can be adapted to other experiments as well. For instance, techniques have been developed to specifically sequence the 5′ region of RNA molecules, allowing the identification of putative transcriptional start sites and helping to define operons and ncRNA [12^••^,13^•^] (Figure 1). In *S.* Typhimurium, coimmunoprecipitation of RNA molecules with Hfq, a chaperone that facilitates hybridization between ncRNA and mRNA, was used to enrich a sample for transcripts participating in such interactions [18^•^], while in *Vibrio cholerae*, a very stringent depletion and size-selection process was used to specifically sequence small ncRNA [23]. RNA-seq has also been applied to whole environments, leading to the development of techniques for sampling the metatranscriptomes of marine [24,25] and soil communities [26].

## Analysis of sequence data

Illumina, 454 and SOLiD sequencing platforms have been used in bacterial RNA-seq studies [27–29]. Each offers a different compromise between the length of reads, which determines what proportion of the genome data can be uniquely mapped to, and depth of coverage, which determines the dynamic range over which gene expression can be quantified.

However, above a certain threshold, obtaining longer reads results in a relatively small increase in the amount of the genome that can be studied, hence read depth will be the more important consideration in almost all cases.

**Figure 2:**
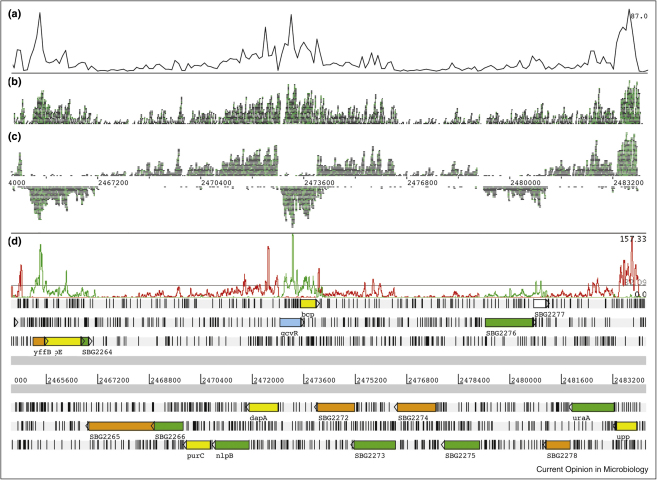
Display of RNA-seq data. Data from a *Salmonella bongori* transcriptome, prepared as described in Ref. [9], displayed using Artemis. Using BamView, the total coverage is shown displayed as a plot **(a)**, as raw reads aligned against the reference sequence **(b)** and as reads assigned separately to the two strands of the genome **(c)**. A strand-specific coverage plot is also shown **(d)** and the genome annotation is displayed underneath.

After sequencing, reads can be assembled using software either based on overlap graphs, such as EDENA [30], or de Bruijn graphs, for instance ABySS [31], ALLPATHS [32] or Velvet [33], which features a strand-specific assembly mode. Alternatively, the reads can be mapped onto a reference sequence. Some studies have used BLAST-based or nucmer-based algorithms [34,35] to align sequence reads to the genome, but a number of programs have been developed specifically for mapping short read data [36–39], which often have the advantages of considering base quality and read pair information when performing alignments. The results of mapping analyses have commonly been visualized as a graph of sequence read coverage across a genome, displayed using software such as the Integrated Genome Browser [40] or Artemis [41]. With the introduction of specialist tools such as BamView [42], raw sequence data can be visualized as well as coverage graphs, allowing a more intuitive understanding of the transcriptional landscape (Figure 2).

RNA-seq, as with comparable methods, requires biological replicates for robust quantification of differential expression. However, the greater cost of sequencing relative to microarray hybridization makes such repetition expensive, so statistical methods have been developed to overcome this by modeling the expected distributions of sequence reads mapping to a locus in different samples. DEGseq [43] uses a Poisson distribution to model the variation between datasets [44], whereas the approaches of edgeR [45] and DEseq [46] are based on the negative binomial distribution, which is suggested to be more appropriate for modeling the variation inherent between biological replicates [47].

## Characteristics of bacterial transcriptomes

The results of bacterial RNA-seq studies have done much to refine our understanding of bacterial gene expression. One initial insight was that genome-wide CDS expression levels appear to be continuously distributed, with no obvious division between actively expressed genes and a ‘background’ transcription level [6,7]. By contrast, marine metatranscriptome studies have found that gene sequences that are most highly represented in cDNA samples are often rare, or absent, from the corresponding genomic DNA samples, suggesting some bacteria may be transcribing a set of uncharacterized genes at an unusually high level [24,25].

Annotation of CDSs has been significantly improved using RNA-seq data. Novel CDSs have been identified in most studies [7–9,11^•^,13^•^,17^•^], including that of *M. pneumoniae*, which has a genome just 816 kb in size [16^••^]. Existing gene models have been refined, often involving correcting the choice of start codon, and associated with one another into operons, which can include the identification of untranslated regions.

However, in both *M. pneumoniae* and *H. pylori*, annotation of transcriptional units was complicated by an unexpectedly high level of flexibility in the structure of operons [12^••^,16^••^]. Evidence from both tiling microarray and RNA-seq data indicated different promoters appeared to be driving expression of the same genes under different conditions, leading to the division of genes into ‘suboperons’. The level of such alternative transcript forms in *M. pneumoniae* was estimated to be similar to that in some eukaryotes [16^••^].

All these amendments to genome annotation are aided by having information on the 5′ ends of transcripts; in *Sulfolobus solfataricus*, mapping these ends was also used to detect putative transcript degradation products. Enrichment of such sites was found to inversely correlate with the half life of the RNA molecule, suggesting an endoribonucleolytic cleavage mechanism may be important in gene regulation [13^•^].

Bacterial whole transcriptome studies have thus far had a very high success rate of ncRNA discovery. Such transcripts have even been identified and mapped to genomes from marine metatranscriptome data, where certain putative ncRNA showed distinct spatial distributions throughout the water column [48]. Validation using RT-PCR and Northern blots has been largely successful [6,12^••^,16^••^,18^•^,23], and work has even begun on functionally characterizing these targets. In *H. pylori*, both *in silico* analysis and mutational inactivation suggested that one novel ncRNA uncovered by RNA-seq regulated a chemotaxis receptor as an antisense RNA [12^••^], and a similar mechanism was posited for a novel ncRNA in *V. cholerae*, which was found to down regulate mannitol metabolism [23].

Directional RNA-seq data are particularly helpful in annotating ncRNA, as it allows reads to be assigned to a particular strand. Furthermore, it has allowed the detection of large amounts of *cis* antisense ncRNA: regions of CDSs that are bidirectionally transcribed, and suggested to act to block expression of the encoded protein [12^••^,13^•^,16^••^,17^•^]. Such transcripts, identified from both whole genome RNA-seq and on the basis of transcriptional start site identification, have been detected and characterized before [49], but the genome-wide scale of their prevalence is only now being appreciated.

Overall, bacterial transcription is starting to appear to more closely mirror that of eukaryotes. Rather than operons being fixed polycistronic transcriptional units, they may represent one way, of several, of transcribing a particular gene, with CDSs having a greater than expected level of independence from their neighbors. Additionally, antisense RNAs, acting either in *cis* or *trans*, may prove to be much more important than previously appreciated.

## Limitations, problems and future directions

RNA-seq datasets have proved to be highly consistent, when comparing either technical or biological replicates, making them appropriate for expression studies [10,50]. However, there are technical issues still awaiting resolution, such as the highly variable nature of the coverage across genes and operons, thought to be the combined result of transcript secondary structure and biases introduced through random hexamer priming of reverse transcription and second strand synthesis [4,51]. This variability, which is generally reproducible between replicate experiments [11^•^], introduces uncertainty into the quantification of RNA abundance. More even coverage has been achieved in eukaryotic datasets through reducing transcript secondary structure by using metal ion-catalyzed hydrolysis to fragment the RNA before reverse transcription [4], and it has been demonstrated that bacterial RNA can be fragmented in a similar manner [5,12^••^,15,17^•^]. There is also the issue of the PCR amplification stages of sequence library construction for all three second generation sequencing platforms, which result in redundant sequence reads and bias the final dataset.

To circumvent such issues, techniques such as direct RNA sequencing [52] and FRT-seq [53], which sequence RNA directly without cDNA intermediates, have been developed. These promise to eventually replace current methods, but suffer from the disadvantage of requiring ribonuclease-free sequencing environments, difficult to maintain in a high throughput sequencing facility. Efforts are also being made to reduce the quantity of starting material required for RNA-seq, with the aim of characterizing the transcriptomes of individual cells [54].

## Conclusions

RNA-seq promises to gradually replace microarrays in most, if not all, genome-wide gene expression studies. Both technologies have their own limitations, but the opportunity to quantitatively study transcription to single nucleotide resolution makes RNA-seq increasingly attractive as sequencing become cheaper and easier. The use of protocols that sequence RNA in a strand-specific manner, and identify transcriptional start sites, will prove especially useful in the identification of ncRNA and defining the operons to which genes belong. Hence there is the potential for this technique to greatly refine our understanding of bacterial gene regulation.

## References and recommended reading

Papers of particular interest, published within the period of review, have been highlighted as:

• of special interest
•• of outstanding interest
